# Comparison of speech perception in bimodal cochlear implant patients with respect to the cochlear coverage

**DOI:** 10.1007/s00106-023-01327-5

**Published:** 2023-08-22

**Authors:** Tobias Rader, Leonhard Schrank, Jennifer L. Spiegel, Pascal Nachtigäller, Judith E. Spiro, John-Martin Hempel, Martin Canis, Joachim Mueller

**Affiliations:** 1 Division of Audiology, Department of Otorhinolaryngology, LMU University Hospital, LMU Munich, Marchioninistr. 15, 81377 Munich, Germany; 2 Department of Otorhinolaryngology, LMU University Hospital, LMU Munich, Munich, Germany; 3 German Center for Vertigo and Balance Disorders, Ludwig-Maximilians-Universität München, Munich, Germany; 4 Department for Radiology, LMU University Hospital, LMU Munich, Munich, Germany

**Keywords:** Hearing aids, Prostheses and implants, Computed tomography, Speech intelligibility, Speech audiometry

## Abstract

**Background:**

The hearing success of patients with bimodal fitting, utilizing both a cochlear implant (CI) and a hearing aid (HA), varies considerably: While some patients benefit from bimodal CI and HA, others do not.

**Objectives:**

This retrospective study aimed to investigate speech perception in bimodally fitted patients and compare it with the cochlear coverage (CC).

**Methods:**

The CC was calculated with the OTOPLAN software, measuring the cochlear duct length on temporal bone CT scans of 39 patients retrospectively. The patients were categorized into two groups: CC ≤ 65% (CC^500^) and CC > 65% (CC^600^). Monaural speech intelligibility for monosyllables at a sound pressure level (SPL) of 65 dB in a free-field setting was assessed before and after CI at various time points. The two groups, one with preoperative HA and one with postoperative CI, were compared. Additionally, speech intelligibility was correlated with CC in the entire cohort before CI and at the last available follow-up (last observation time, LOT).

**Results:**

Overall, there was no significant difference in speech intelligibility between CC^500^ and CC^600^ patients, with both groups demonstrating a consistent improvement after implantation. While CC^600^ patients tended to exhibit earlier improvement in speech intelligibility, CC^500^ patients showed a slower initial improvement within the first 3 months but demonstrated a steeper learning curve thereafter. At LOT, the two patient groups converged, with no significant differences in expected speech intelligibility. There was no significant relationship between unimodal/unilateral free-field speech intelligibility and CC. Interestingly, patients with a CC of 70–75% achieved the highest speech intelligibility.

**Conclusion:**

Despite of the lack of a significant correlation between CC and speech perception, patients appeared to reach their maximum in unimodal/unilateral speech perception primarily at a coverage level of 70–75%. Nevertheless, further investigation is warranted, as CC^500^ was associated with shorter cochlear duct length, and different types of electrodes were used in both groups.

## Physioanatomical characteristics

Paramount for the success of cochlear implants (CI) is the individualized care of each patient The CI team takes into account the diverse (patho)physioanatomical characteristics of each patient, including inner ear malformations [1], residual hearing [2, 3], prevention of vertigo [4], and the wide range of cochlear duct lengths (CDL; [5–7]). Different CI manufacturers offer a selection of electrodes with carrying lengths and locations within the cochlea [8–11]. The modality should also be considered when fitting both ears.

### Modality categories

A substantial proportion of CI recipients fall into one of the following five categories: (1) Patients with single-sided deafness (SSD) who have normal hearing in one ear and a CI in the other ear [12–14]. (2) Bimodally fitted patients with asymmetric hearing, fitted with CI in one ear and a contralateral hearing aid (HA) in the better, not CI-indicated ear [12]. (3) Bimodally fitted patients who meet the indication criteria for a CI on both ears but are fitted with CI in the worse and HA in the better-hearing ear [15]. (4) Patients with electroacoustic stimulation (EAS) in one ear and a HA in the other ear. These patients have good low-frequency residual hearing and receive an HA integrated into the CI audio processor, which allows simultaneous electrical and acoustic stimulation from the CI processor itself [3, 16]. (5) Bilaterally deaf patients fitted with bilateral CIs [17].

### Variability in binaural integration

However, within these categories there are considerable differences in the degree of binaural integration. Some patients with bilateral fittings experience substantial benefit from binaural hearing, while others experience minimal to no benefit. Disadvantages, such as binaural interference, are also possible. Several individual characteristics contribute to this variability, including residual hearing preservation [18], cortical plasticity, duration of deafness [19], differing processing times of CI and HA [20, 21], frequency discrepancy between CI and HA ear [22, 23], and differences in automatic gain control between CI and HA [24]. Bimodal interference is observed in some patients, who report better hearing when using only one ear [25, 26]. Another factor that may influence the success of bimodal fitting is the cochlear coverage (CC) provided by the electrode array. The question is whether a monaural CI with a larger CC can offer better low-frequency hearing and, thus, avoid interference with the contralateral side supplied by the HA. Therefore, the aim of the study was to investigate monaural speech perception in bimodally fitted patients with CI and HA with respect to the CC.

## Materials and methods

### Patients

A retrospective, single-center analysis was performed comprising a total of 39 bimodally fitted patients. Pre- and postoperative audiometric data, as well as the radiological Stenverʼs view to assess CI electrode placement (complete full insertion in all patients), were available for evaluation. Patients were implanted with either FLEX28 (28 mm, active stimulation length 23.1 mm) or FLEXSOFT electrodes (31.5 mm, active stimulation length 26.4 mm) from MED-EL (Innsbruck, Austria; [27]).

### Cochlear coverage

The CC was determined in all patients using the OTOPLAN software (CAScination AG, Bern, Switzerland, version 2) based on CT images (CE certification number: G1 17 10 95657 003). During preoperative planning, the software enables measurement of the cochlea using Digital Imaging and Communications in Medicine® (DICOM®) datasets, to determine the insertion depth of the CI electrode array and the CC [28].

All DICOM® datasets were initially reviewed for image quality and temporal bone malformations by a radiologist experienced in temporal bone anatomy before being uploaded into the software. Exclusion criteria were cochlear malformations, CT slice thickness of ≥ 0.7 mm, and datasets that could not be transferred to the OTOPLAN software for technical reasons.

The cochlea was measured preoperatively in three planes using the software described in detail elsewhere [7]. In brief, the software calculated the length of the cochlear duct using an elliptical-circular approximation (ECA) based on the measured values of “A-value” (maximum distance between the round window and the contralateral wall), “B-value” (distance between the walls of the cochlea perpendicular to the line of the A‑value), and “height” (distance perpendicular to the basal turn of the cochlea to the apex; [29]). The CC was then determined computationally using the expected angular insertion depth (AID) from the preoperative CT dataset for the selected electrodes, and the frequency-location mapping in the cochlea was estimated using the Greenwood function based on selected electrodes [29]. A CC of 100% corresponded to 2.5 turns of the cochlea, resulting in an AID of 900° [30]. All measurements were performed by two independent examiners blinded to each other’s measurements and electrode information, and the resulting measurement results were averaged.

For further analysis of CC, participants were divided into two comparator groups of approximately equal size, regardless of electrode type. One group had a CC of ≤ 65% (mean AID of this cohort: 498.6°; designated CC^500^) and the other group a CC of > 65% (mean AID of this cohort: 591.1°; designated CC^600^).

### Audiometric data

To perform hearing threshold audiometry, sinusoidal tones standardized according to German DIN EN ISO 8253 were presented successively in different frequencies between 0.250 and 8 kHz. This was done under both air conduction and free-field conditions in an audiometrically isolated booth. If necessary, the non-tested ear was additionally masked with noise according to the guidelines of the *Comité Consultatif International Télégraphique et Téléphonique* (CCITT) to prevent cross-over hearing on the opposite ear. The tones were presented through headphones unaided for air conduction; for free field they were presented through a loudspeaker to assess separately the aided hearing threshold with HA/CI (inflation curve) side by side. The resulting pre- and postoperative thresholds, measured in decibel hearing level (dB HL), were compared and documented. For the postoperative values, the most recent hearing status data from the patient’s medical record, referred to as the last available observation time (LOT) for follow-up, was used.

Speech intelligibility, measured with the German Freiburg Speech Test standardized according to German DIN 45621‑1 and DIN 45626‑1 at a sound pressure level (SPL) of 65 dB [31], was obtained retrospectively from electronic medical records.

Monaural monosyllable aided speech perception was assessed in the implanted ear, preoperatively with HA and postoperatively with CI (‑audio processor). Postoperative testing was performed at the time of initial fitting (IF), 1 month (1M), 3 months (3M), and 1 year (12M) after initial fitting, and at LOT.

### Statistical analysis

Statistical analysis of the data was performed using Microsoft Excel (Microsoft, Redmond, WA, USA, version 2110) and the Statistical Package for Social Sciences (SPSS) software (IBM, Armonk, NY, USA, version 28).

For normally distributed data, the unpaired samples *t *test was used to compare the means of CC and several cochlear morphology parameters (CDL, A‑value, B‑value, and AID), and speech intelligibility between the CC^500^ and CC^600^ groups. In the case of non-normal distributions, the Mann–Whitney *U *test was performed to compare the median values of cochlear height and speech intelligibility between the CC^500^ and CC^600^ groups. The correlation between speech perception for monosyllables and CC was also examined using the Pearson correlation coefficient. The significance level was set at 0.05.

## Results

### Demographics

The median age of the 39 patients enrolled in the study at the time of implantation was 65 years (15–90 years). Of these patients, 27 received a FLEX28 electrode and 12 a FLEXSOFT electrode. Table 1 provides an overview of the etiology of profound sensorineural hearing loss leading to deafness in the patient population.

**Table 1:** Most common etiologies of the study participants study

Etiology	n
Meniere’s disease	6
Hearing loss	3
Heredity	3
Intracochlear schwannoma	2
Large vestibular aqueduct syndrome with Mondini malformation	1
Unknown	24
Total	39

### Cochlear coverage

Of the 39 patients, 14 were in the CC^500^ group and 25 were in the CC^600^ group. The mean CC for the CC^500^ group was 60.6 ± 3.6%, while the CC for the CC group^600^ was 73.1 ± 5.4%. The mean CC for the entire cohort was 68.6 ± 7.7%. Table 2 shows additional parameters such as CDL, A and B values, height, and AID. The *t* tests revealed significant differences between the C^500^ and CC^600^ group for CC (*t*(37) = −8.61; *p* < 0.001), CDL (*t*(37) = 3.67; *p* *=* 0*.*001), A‑value (*t(37)* *=* 2.74; *p* *=* 0.009), B‑value (*t(37)* *=* 3.77; *p* *=* 0*.*001), and AID (*t*(37) = −5.96; *p* < 0.001). However, there was no significant difference regarding height (*U* = 116.50; *Z* = −1.72; *p* > 0.05).

**Table 2:** Mean ± standard deviation and *p *values of *t *tests for cochlear morphological parameters of CC^500^ and CC^600^ groups and the entire cohort

Morphology	CC^500^ (*n* = 14)		CC^600^ (*n* = 25)		*p*	Total cohort (*n* = 39)	
CC (%)	60.6	±3.6	73.1	± 5.4	0.000^*^	68.6	±7.7
CDL (mm)	37.2	±1.4	35.0	± 2.0	0.001^*^	35.8	±2.1
A‑value (mm)	9.6	±0.4	9.2	± 0.4	0.009^*^	9.4	±0.4
B‑value (mm)	7.3	±0.3	6.7	± 0.5	0.001^*^	6.9	±0.5
Height (mm)	4.3	±0.2	4.2	± 0.4	0.324	4.2	±0.3
AID (°)	498.6	±29.5	591.1	±53.5	0.000^*^	557.9	±64.2

*AID* angular insertion depth, *CC* cochlear coverage, *CDL* cochlear duct length, *n* number, *SD* standard deviation

*Significant values

### Audiometric data

**Fig. 1:**
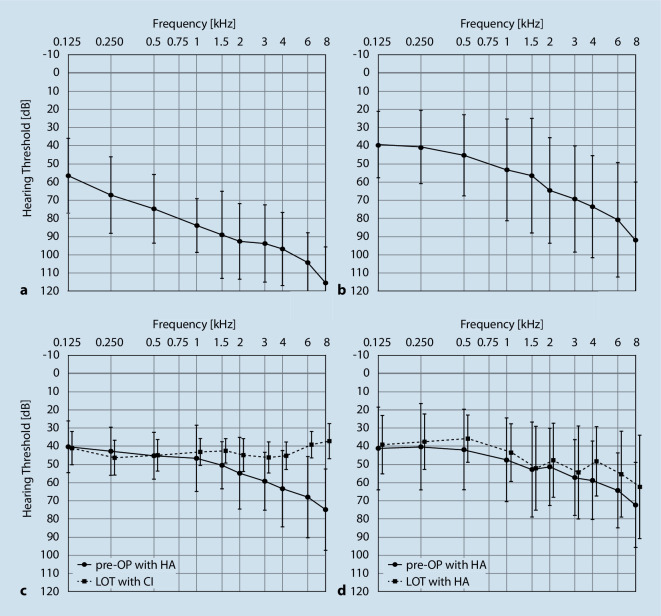
Means and standard deviations of preoperative unaided air conduction thresholds (**a**, **b**) and pre-/postoperative (*last observation time, LOT*) aided threshold curves with hearing aid (*HA*) and cochlear implant (*CI*), respectively (**c**, **d**)

Audiometry was performed on 39 bimodally fitted patients using air conduction and free-field audiometry. Figure 1a shows the consistent decrease in air conduction hearing threshold for the implanted ear preoperatively without HA from 57 dB HL at 125 Hz to 115 dB HL at 8 kHz, with a pure tone average (PTA; 0.5; 1; 2; 4 kHz) of 87.4 dB HL. Similarly, preoperative air conduction for the nonimplanted contralateral ear showed a consistent decrease from 39 dB HL at 125 Hz to 92 dB HL at 8 kHz, resulting in a PTA of 59.3 dB HL (Fig. 1b).

For the implanted ear, the free-field hearing threshold showed a decreasing trend along with increasing frequency with HA preoperatively, ranging from 40 dB HL at 125 Hz to 75 dB HL at 8 kHz (PTA = 53.2 dB HL; Fig. 1c). With the CI at LOT, the hearing thresholds were similar across frequencies, ranging from 39 dB HL at 125 Hz to 37 dB HL at 8 kHz (PTA = 44.7 dB HL; Fig. 1c). The preoperative PTA for the HA-equipped contralateral ear averaged 49.9 dB HL, and the PTA at the LOT averaged 43.9 dB HL (Fig. 1d). Additional pure-tone audiometric data for the implanted ear at LOT are presented in Table 3.

**Table 3:** Medians and ranges of PTA (0.5; 1; 2; 4 kHz) in the free field with the CI and speech perception for monosyllables at 65 dB SPL with the CI for the CC^500^ and CC^600^ groups and the entire cohort

	CC^500^ (*n* = 14)		CC^600^ (*n* = 25)		Total cohort (*n* = 39)	
	Median	Range	Median	Range	Median	Range
PTA attended hearing threshold (dB HL)	46.25	36.25–58.75	43.00	30.00–62.50	43.75	30.00–62.50
Speech intelligibility (%)^a^	65	0–90	60	15–95	60	0–95

*CC *cochlear coverage, *CI *cochlear implant, *dB* decibels, *HL* hearing level, *PTA* pure tone average for 0.5, 1, 2, and 4 kHz, *SPL* sound pressure level

^a^Speech intelligibility for monosyllables at 65 dB SPL

**Fig. 2:**
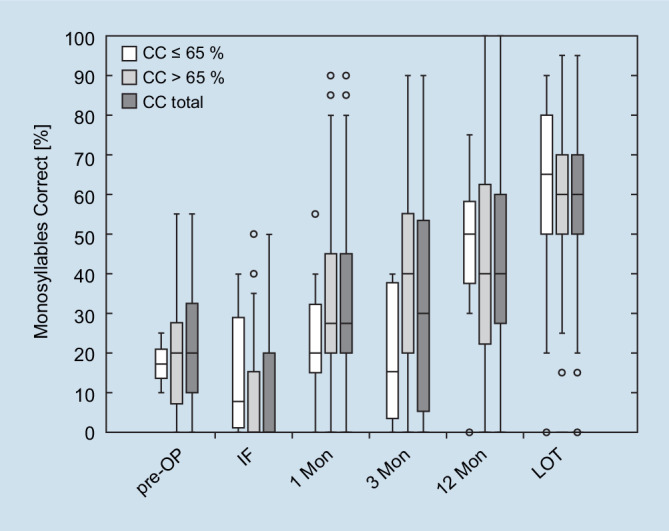
Box–whisker plots of speech perception for monosyllables at 65 dB SPL in the groups of patients with cochlear coverage (CC) of CC ≤ 65% (CC^500^), CC > 65% (CC^600^), and the entire cohort (CC total = CC^500^ + CC^600^) at different observation times: preoperatively (*pre-OP*), initial fitting (*IF*), 1 month (*1* *Mon*), 3 months (*3* *Mon*), and 12 months (*12* *Mon*) after IF, and at the last available observation time point (*LOT*). Outliers are marked with *+*

Regarding the assessment for normality, all datasets, except for monosyllable intelligibility of the C^600^ group, displayed normality at the IF and at the 1M appointment (Shapiro–Wilk *p* < 0.001 and *p* = 0.02). The *t *tests and Mann–Whitney *U *test results revealed no significant differences in speech intelligibility between CC^500^ and CC^600^ at the observed time points. Figure 2, using box–whisker plots, illustrates a trend in which speech perception in the implanted ear initially appeared to deteriorate from 20% of the group median at the preoperative measurement to 0% at the IF, but showed steady improvement postoperatively with the CI at the subsequent measurement time points: 1M after IF (25%), 3M (30%), 12M (40%), and at LOT (60%). At IF, patients in the CC^500^ group initially appeared to perform better in speech perception (7.5%) than patients in the CC^600^ group (0%); however, this difference was not significant. However, at the 1M appointment, patients with CC^500^ showed a slight reduction in speech perception (20%) compared to those with CC^600^ (27.5%). At 3M, patients with CC^500^ (15%) appeared to have worse speech perception than those with CC^600^ (40%). However, between the 3 and 12M appointments, patients with CC^500^ (50%) appeared to have greater improvement in speech perception compared to those with CC^600^ (40%). At LOT, the two groups converged in speech perception. Additional speech perception data with the implanted ear at LOT are presented in Table 3.

### Correlation of cochlear coverage with speech perception

**Fig. 3:**
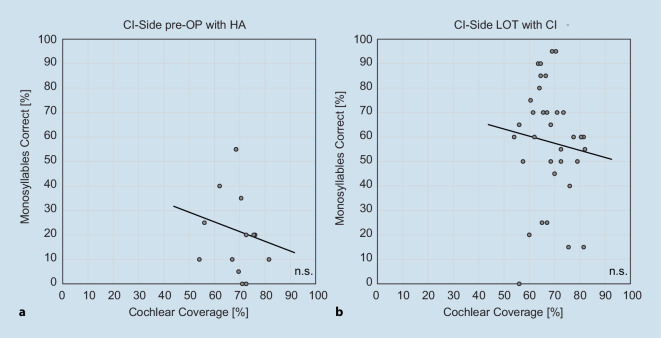
Scatter plot, regression line (*dashed* *line*), and the results of correlation analysis (*r*, *p*) between cochlear coverage and speech perception at 65 dB SPL on the implanted side preoperatively with hearing aid (*HA;* **a**) and at the last available observation time point (*LOT*) with cochlear implant (*CI*; **b**)

Correlation analysis revealed no significant correlation between total CC (total cohort of CC^500^ and CC^600^) and intelligibility at 65 dB SPL, neither preoperatively monaurally with HA (*n* = 14; *r* = −0.16; *p* > 0.05) nor postoperatively with a CI at LOT (*n* = 34; *r* = −0.09; *p* > 0.05). Figure 3 illustrates that patients with approximately 70–75% CC achieved maximum speech intelligibility within the entire cohort.

## Discussion

### The present study

The primary objective of this study was to investigate the correlation between CC in bimodally fitted patients (CI + contralateral HG) and monaural speech perception in the implanted ear.

No significant correlation between CC and monaural speech perception was observed at any time point for the entire group of patients.

Interestingly, contrary to our assumption that complete CC (CC = 100%, corresponding to an AID of 900° or an insertion of 2.5 turns) would result in better speech perception, we found a maximum in speech perception at approximately 70–75% CC. However, due to the large interindividual variability in speech perception, the low nonsignificant coefficients, and the small sample size, these results should be interpreted with caution. Future studies with a larger patient cohort are needed to validate the hypothesis of a maximum at 70–75% CC. It is also important to include patients with 50–60% CC and 80–90% CC, as they were underrepresented in this study. This may shed light on the possible nonlinear relationship between CC and speech perception. The effect of preoperative residual hearing on speech perception and its association with CC was not thoroughly investigated in this study. Further research is warranted to investigate the influence of residual hearing on speech perception outcomes. In addition, within-subject variability in speech intelligibility was observed at different test time points, which may be due to various psychological/behavioral factors such as motivation or concentration. These factors further complicate the interpretation of the results.

Nevertheless, our results show that the CC^500^ and CC^600^ groups converge at the LOT with similar medians and dispersions after different levels of speech perception with electrical stimulation by CI. It is worth noting that the shorter CC^500^ group initially required a longer learning period for speech perception.

### Comparison with other studies

Previous studies have not reached a consensus on whether there is a relationship between CC of the electrode array and monaural speech perception with CI. For example, Doubi et al. [32] divided prelingually deafened children under 7 years of age into two groups and found no significant difference in speech perception, measured by the speech intelligibility rating test, between a group with CC < 85% and a group with CC ≥ 85% at 3 years postoperatively. They concluded that stimulation of the most apical region of the cochlea does not necessarily provide a benefit for speech perception. Other studies have used a metric comparable to CC, the angular insertion depth (AID), and examined its relationship to speech perception. The majority of these studies reported no correlation between speech perception and AID [19, 20, 25, 37, 42, 43]. Heutink et al. [33] conducted a comprehensive systematic review of previous studies and found no significant correlation in six out of seven studies [34–39]. However, other studies have shown a relationship between AID and speech perception: O’Connel et al. [40] observed a significant positive correlation in postlingually deafened adults, measured 12–16 months postoperatively, with a 0.6% increase in the English Consonant Nucleus Consonant (CNC) score at 60 dB (A) per 10° AID. Similar positive correlations were found by Canfarotta et al. [41] using the CNC score of adult unilateral CI recipients measured 12 months after IF and by Heutink et al. [42] using the Dutch Consonant–Vowel–Consonant test measured in adult CI users with at least 1 year of unilateral hearing experience. By contrast, Ketterer et al. [43] examined adult CI recipients with the Freiburg Monosyllabic Test at 65 dB SPL at regular intervals and found a significant negative relationship indicating a decrease in speech perception with increasing insertion depth. Although we did not find a significant relationship between insertion depth and monaural speech perception in our study, larger insertion depths (> 75%) appeared to have a negative effect on speech perception. This could be explained by the possibility that deeper insertions are more likely to damage any residual hearing that may be present. Ketterer et al. [43] attributed their results to cross-turn stimulation, which may occur with deeply inserted apical electrodes. However, deep insertion was underrepresented in the study by Ketterer et al. [43], with only approximately 2% of the ears included (10 out of 495) having a CC > 75%. In addition, better preoperative speech perception may lead to better postoperative speech perception with CI.

An advantage of electrical stimulation at the apex of the cochlea is improved perception of low frequencies, which are also particularly important for music perception. When listening to music, a mixed cohort of bilateral and SSD CI users with longer electrodes (31.5 mm) experienced better perception of lower frequencies compared to users with 7.5 mm shorter electrodes (24 mm) due to the more extensive apical stimulation [44]. This results in improved discrimination of sound quality for patients with long electrode arrays.

Speck et al. [38] conducted a study to investigate the effects of different electrode lengths (active stimulation length: 15.0 mm versus 19.1 mm versus 23.1 mm) on speech reception thresholds (SRT) in SSD patients. Assessment of SRT was made under two different noise conditions: speech and noise collocated at the front (S0N0) and speech on the implanted side with masking noise on the normal-hearing side. In both conditions, no significant difference in SRT was found between the electrode array lengths.

Deep insertion may provide a more natural hearing experience, especially for bimodal CI users with an HA on the contralateral side, as well as for SSD CI users, because the electrodes of the lower frequencies are closer to the apical spiral ganglion cells that correspond to these low frequencies according to Greenwood’s frequency-location mapping in the cochlea [30]. Presumably, this is the reason why patients with a deeper insertion in this study adapted more quickly to hearing with the CI and went through the learning process faster than those with a shallower insertion, because the sound field perceived over the CI was less pitch shifted. Whether this holds true for all postlingually deafened patients remains to be tested.

## Practical conclusion

- We did not find a significant correlation between monaural monosyllabic speech perception with a cochlear implant (CI) and the cochlear coverage (CC) provided by the CI electrode in bimodally (CI and hearing aid [HA]) fitted patients.
- A trend was observed: speech perception increased with increasing CC, reaching a maximum at ca. 70–75% and then decreased with further increases in CC.
- The lack of a significant relationship may be due to the large variability in a small patient population, resulting in insufficient statistical power, as well as the possibility of a nonlinear relationship between CC and speech perception. Linear Pearson correlation analysis was not suitable for assessing the possibility of a nonlinear relationship, partly due to the covariance of the electrode array (FLEX28, FLEXSOFT).
- Patients with a greater insertion depth achieved faster learning success. Despite different CC^500^/CC^600^ mean values (59.2 ± 28.4%/47.1 ± 21.9%) and medians (65%/60%) of the CC, no significant difference in long-term speech perception was found.
- In clinical practice, preoperative measurement of the cochlea and individualized electrode selection are beneficial to determine the most suitable electrode length for every patient.
- A CC of 70–75% was identified as a good reference point for CC, but more research is needed in this area before a definitive recommendation can be made.

## Declarations

All procedures performed in studies involving human participants or on human tissue were in accordance with the ethical standards of the responsible ethics committee of the Ludwig-Maximilians-University of Munich (reference 19-562) and with the 1975 Helsinki declaration and its later amendments or comparable ethical standards. Informed consent was obtained from all individual participants included in the study.
